# Hepatitis C Virus Evasion from RIG-I-Dependent Hepatic Innate Immunity

**DOI:** 10.1155/2010/548390

**Published:** 2011-01-17

**Authors:** Helene Minyi Liu, Michael Gale

**Affiliations:** Department of Immunology, School of Medicine, University of Washington, Seattle, WA 98195-7650, USA

## Abstract

Exposure to hepatitis C virus (HCV) usually results in persistent infection that often develops into chronic liver disease. Interferon-alpha (IFN) treatment comprises the foundation of current approved therapy for chronic HCV infection but is limited in overall efficacy. IFN is a major effector of innate antiviral immunity and is naturally produced in response to viral infection when viral pathogen-associated molecular patterns (PAMPs) are recognized as nonself and are bound by cellular pathogen recognition receptors (PRRs), including Toll-like receptors (TLRs) and the RIG-I-like receptors (RLRs). Within hepatocytes, RIG-I is a major PRR of HCV infection wherein PAMP interactions serve to trigger intracellular signaling cascades in the infected hepatocyte to drive IFN production and the expression of interferon-stimulated genes (ISGs). ISGs function to limit virus replication, modulate the immune system, and to suppress virus spread. However, studies of HCV-host interactions have revealed several mechanisms of innate immune regulation and evasion that feature virus control of PRR signaling and regulation of hepatic innate immune programs that may provide a molecular basis for viral persistence.

## 1. Introduction

In response to virus infection, signaling pathways within mammalian cells direct a variety of intracellular events that generate an antiviral state directly within the infected cell. This antiviral innate immune response represents our very frontline of immune defense against virus infection. If this response is successful, exposure to the virus will turn into an abortive, self-limiting infection, and the virus will be cleared. It is the hepatic innate immune response that imposes initial antiviral defenses against HCV infection [1]. This response is triggered when the infected cell recognizes a molecular signature or PAMP within the invading virus through the actions of cellular PRRs [2, 3]. The PAMP/PRR interaction initiates signaling cascades that induce the expression of antiviral effector genes [4, 5]. Viral protein and/or nucleic acid products comprise an array of PAMP signatures that can engage specific PRRs, including TLRs or the RLRs, RIG-I, and MDA5 [6–9].

**Figure 1:**
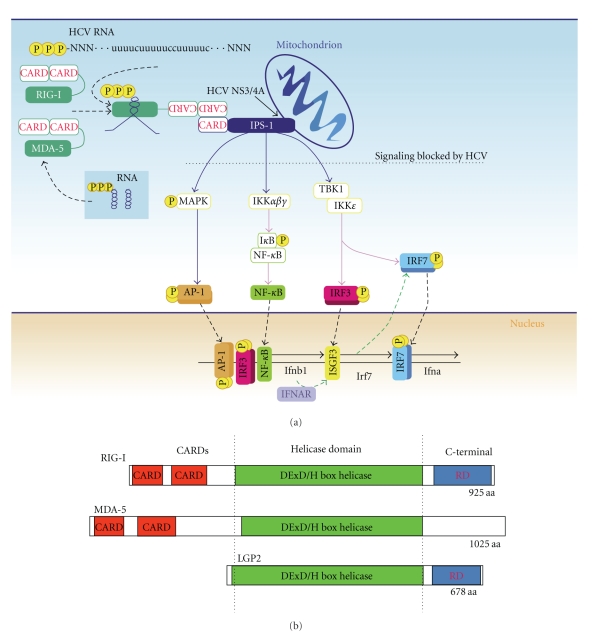
(a) The innate immune induction pathway through RLR activation. HCV 5′ppp RNA with poly-U/UC motif is shown as the RIG-I ligand RNA; 5′ppp dsRNA is depicted beneath MDA5 and could serve as either RIG-I or MDA5 ligand RNA. The site of NS3/4A targeting of IPS-1 within the RLR pathway during HCV infection is indicated by the arrow, and the resulting signaling blockade is indicated by the broken line. (b) The RLR family members. CARD, helicase, and C-terminal domains, including the repressor domain (RD), are indicated. Numbers refer to amino acid (aa) length.

Upon virus infection and PAMP recognition, RLRs and TLRs operate through independent signaling cascades separated in part through the localization of the distinct PRRs. RLRs are cytosolic whereas TLRs are typically localized within endosomes or on the cell surface. Nevertheless, both sets of PRR signaling cascades can lead to the activation of PAMP-driven transcription factors, IFN gene expression and protein secretion, and ISG expression that results in the immediate induction of the intracellular innate immune response both within the infected cell and within bystander cells that respond locally or systemically to the secreted IFN [10]. Interferon regulatory factor- (IRF-) 3 and nuclear factor-kappa B (NF-*κ*B) are the first transcription factors activated in response to HCV infection of hepatocytes (Figure 1(a)). During HCV infection, their activation proceeds through RIG-I signaling, with likely contributions from TLRs, whose signaling pathways promote IRF-3 and NF-*κ*B nuclear translocation and transactivation functions. Other IRF family members, including IRF-1, IRF-5, and IRF-7, contribute to innate immune responses and should be considered important for immunity against HCV infection [11, 12].

## 2. Toll-Like Receptors Mediate Endosomal PAMP Recognition of HCV

Toll was first identified as a transmembrane receptor regulating insect morphogenesis [13]. Toll mutation also results in increased susceptibility to fungi in Drosophila [14]. Ten members of human Toll-like receptors (TLRs) were later identified as sensing receptors of various pathogen-associated molecular patterns (PAMPs). Human TLRs are expressed in a tissue-specific manner, and many are expressed in dendritic cells (DCs) and macrophages [15]. Although each TLR detects a distinct set of PAMPs, a common extracellular leucine-rich repeat (LRR) motif is responsible for PAMP sensing. When the LRR engages a PAMP, the TLR transmits a signal through the cytoplasmic domain of the receptor to drive a signaling cascade that results in the production of various cytokines that serve to define the innate immune response and initiate immune cell recruitment.

This signaling drives macrophages and DCs to differentiate into full-blown antigen-presenting cells to initiate antigen-specific adaptive immunity. In humans, at least three major TLRs are important in virus infection and immunity: TLR3 [16–18], TLR7 [19–24], and TLR9 [25, 26], which are typically expressed within endosomes. Double-stranded RNA (dsRNA) PAMPs are detected by TLR3 whereas TLR7 recognizes a specific uridine-rich ribonucleotide motif within a single-strand RNA [27], and TLR9 recognizes DNA PAMP motifs encoding CpG nucleotides [28, 29]. It has also been reported that TLR2 and TLR4 are also involved in inflammation responses during HCV infection [30–32]. Thus, TLR3 and TLR7 specifically recognize PAMPs that accumulate during RNA virus infection, and each has been shown to be important for innate immunity against HCV infection either directly in hepatocytes (TLR3) or indirectly through PAMP signaling of antigen-presenting cells (TLR3 and TLR7) that accumulate HCV products through their phagocytic activity [15, 33, 34].

## 3. RLRs Mediate Cytosolic RNA PAMP Recognition

Retinoic acid-inducible gene-I (RIG-I) is the prototypical member of the RLR family, which also includes melanoma differentiation-associated gene 5 (MDA5) and laboratory of genetics and physiology 2 (LGP2). The RLRs have a C-terminal RNA helicase domain with RNA-binding activity [35]. RIG-I and MDA5 contain N-terminal tandem caspase activation and recruitment domains (CARDs), but these are not present within LGP2 (Figure 1(b)). Both RIG-I and LGP2 are regulated by a C-terminal repressor domain, which remains unidentified in MDA5 [36, 37] (Figure 1(b)). Recent studies have revealed that RIG-I and MDA5 detect different RNA viruses, with RIG-I being essential for detection of HCV [9, 38]. The mechanism of PAMP recognition by RIG-I has been best characterized among the RLRs (presented below). The nature of MDA5 PAMP ligands was recently defined as long-stable double-stranded (ds) RNA that are distinct from RIG-I ligands [39].

## 4. The Role of RIG-I in Nonself Recognition and Antiviral Innate Immunity

Early studies previously defined an association between retinoic acid and ISG expression wherein it was reported that the expression of certain ISGs could be in part induced by retinoic acid [40, 41]. Indeed, in addition to its expression being induced by retinoic acid, RIG-I is an ISG and its PRR function may serve to connect IFN and retinoic acid signaling events that modulate antiviral immunity. RIG-I is the best characterized of the RLRs and was implicated as a PRR through functional cDNA screening that identified human RIG-I as positive regulator of ISG expression [42] and as a PRR of HCV [43]. Structure-function studies have identified the RIG-I CARDs as the signaling domain, which interacts with a downstream molecule, IPS-1, to relay signaling to IRF-3 and NF-*κ*B. Indeed, overexpression of the tandem CARD alone is sufficient to activate downstream signaling and subsequent type I IFN production [35], and the tandem CARD domains are necessary for RIG-I function [36]. In an interferon-cured HCV replicon cell, Huh 7.5, the amino acid T55 within the first CARD domain was found to be mutated to an isoleucine, which associated with loss of innate immune induction by RNA virus infection and increased permissiveness to HCV RNA replication compared to RIG-I wild-type parental cells [43]. This mutation in RIG-I (T55I) has recently been shown to abrogate RIG-I interaction with the E3 ubiquitin ligase, TRIM25, to ablate TRIM25-mediated RIG-I ubiquitination that otherwise serves to enhance RIG-I signaling activation and interaction with the IPS-1 adaptor protein [44].

Understanding the nature of RIG-I ligand/PAMP RNA continues to be a major focus of the innate immunity research field. Our current understanding of RIG-I ligand biology centers on the role of exposed 5′ triphosphate (5′ppp) as the central feature of a nonself PAMP ligand of RIG-I. Various studies have shown that RIG-I can bind single-stranded (ss) or double-stranded (ds) RNA but in each case PAMP recognition is dependent upon the RNA harboring a 5′ppp. For dsRNA, RIG-I preferentially recognizes RNA longer than 25-base pairs with an ssRNA overhang region containing a 5′-triphosphate (5′ppp) motif [35, 38]. While RIG-I does not bind to DNA, it selectively binds to poly(rI:rC) and poly(rA:rU) dsRNA, and poly-U/UC ssRNA, the later identified as a PAMP motif within the HCV genome [42, 43]. These studies showed that cytoplasmic ssRNA containing a 5′ triphosphate and uridine- or adenosine-rich viral RNA motifs of a variety of viruses are well recognized by RIG-I, and that PAMP RNA binding and innate immune signaling are governed by the C-terminus repressor domain (RD) of RIG-I [45].

**Figure 2:**
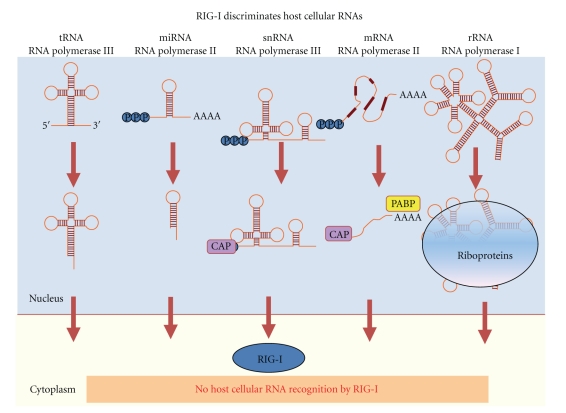
RIG-I discrimination of self and nonself RNA. The primary transcripts of cellular mRNA are produced in the nucleus and are modified before export to the cytoplasm; 5′ppp is replaced by a 5′ cap structure on mRNAs while it is processed from tRNAs; microRNAs are processed to a length insufficient for RLR recognition. Cellular RNA-binding proteins can prevent RIG-I detection of 5′-triphosphatae in rRNAs though masking as an RNP.

How are endogenous “self” and viral “nonself” RNA species actually discriminated by RIG-I? As shown in Figure 2, host RNA synthesis occurs in the nucleus. Unprocessed cellular RNA transcripts contain 5′-triphosphate. However, the 5′-triphosphate is modified or processed before the transcripts arrive to the cytoplasm; the mRNA acquires a 7-methylguanosine CAP structure at its 5′ end; tRNA undergoes 5′ cleavage and a series of nucleotide base modifications; ribosomal RNA, which does possess 5′ppp, is readily complexed with ribosomal proteins to form ribonucleoprotein that masks the 5′ppp from RIG-I recognition. Indeed, 5′-OH or a 5′-methylguanosine capped RNA do not bind to RIG-I and therefore cannot promote the conformational change required for RIG-I activation [36, 45–48]. Endogenous, self RNA species thus avoid detection by RIG-I by the presence of a 5′ cap, specific RNA processing, or compartmentalization as an RNP. Viral RNA, however, either freshly introduced by infection or produced during viral replication, contains the essential nonself marker 5′-triphosphate paired with other nonself motifs such as dsRNA or ssRNA poly-U/UC PAMP motifs. Recently, it is reported that cytosolic poly(dA-dT) DNA motifs are converted into 5′-triphosphate RNA by RNA polymerase III within the host cell cytosol and can thus induce innate immune programs through nonself recognition of the RNA product by RIG-I [49, 50]. This pathway may be important in the sensing of Epstein-Barr virus-encoded small RNAs, which are transcribed by RNA polymerase III. These findings suggest that viral and possibly even certain undefined cellular pol-III transcripts should be considered as possible PAMP RNA ligands of RIG-I.

The overall structural features of the RLRs define each as a DEx/D box RNA helicase. In terms of RIG-I, the helicase domain and C-terminal RD mediate nonself RNA recognition and binding of viral PAMP RNA [42]. RIG-I activation is dependent on PAMP RNA binding and the actions of the RD such that ectopic expression of full-length RIG-I will not render signaling of ISG expression unless the RD engages a specific RNA PAMP. Functional analyses revealed that RIG-I is maintained in an autorepressed state through the RD mediating intermolecular inhibitory interactions with the CARD and helicase domains [36], and that signaling activation occurs upon PAMP RNA binding that repositions the RD and CARDs into a signaling-ON state [36, 51]. RD repositioning is dependent on ATP hydrolysis activity of RIG-I, which also serves to drive RIG-I translocation along a bound RNA to survey for PAMP motifs [52]. By this model, RIG-I signaling activation proceeds once RIG-I has engaged a PAMP motif within a bound RNA to thus confer RD repositioning and release of signaling autorepression. An important feature of this model is that RIG-I would be constantly binding and translocating along an RNA until it encounters a PAMP motif defining specific ligands as nonself or until it releases (self) RNA lacking PAMP features. Thus RIG-I and the RLRs in general may survey the cytosolic environment for nonself, PAMP RNA. In this sense, RIG-I is a constant sentinel poised to rapidly detect HCV infection within the hepatocyte.

## 5. Triggering the Innate Immune Response to HCV Infection

As noted above, the nature of the host cell PRR that serves to detect HCV RNA as nonself and to trigger the innate immune response to HCV infection was revealed through studies of the Huh7-derived cell line, termed Huh7.5. This cell line does not exhibit an intracellular innate immune response to RNA virus infection and was found to be highly permissive to supporting HCV RNA replication [43, 53]. cDNA complementation studies identified RIG-I as a PRR for HCV RNA wherein RIG-I was first thought to bind to HCV dsRNA motifs located within the viral genome 3′ nontranslated region (NTR). These studies revealed that RIG-I was essential for triggering the activation of IRF-3 and NF-*κ*B in response to RNA virus infection in hepatocyte-derived cells, resulting in IFN-*β* expression and onset of the intracellular innate immune response [43]. Furthermore, in cultured cells the HCV NTRs present dsRNA PAMP structures that may serve as potent agonists of TLR3 signaling [33], though this has not been formally proven *in vivo*. Together, these observations suggest that during HCV infection various RNA motifs are recognized and engaged by RIG-I and possibly TLR3 to trigger antiviral defenses [43].

**Figure 3:**
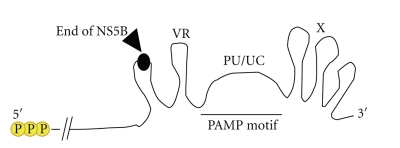
Domains of the HCV genome featuring the 3′ NTR. Details are described in the text. 5′ppp and the poly-U/UC region of the HCV genome or the genome replication intermediate RNA are the major PAMP determinants of HCV that are recognized by RIG-I. Arrow head marks the end of the HCV protein-coding region. The PAMP motif is indicated.

The ability of HCV RNA to trigger innate immune signaling in hepatoma cells and in the liver within *in vivo* mouse model was evaluated using molecular approaches to define the specific PAMP motifs responsible for immune triggering. The outcome of these studies revealed that RIG-I was essential for innate immune signaling in hepatocytes and for hepatic innate immunity triggered by HCV RNA *in vivo*. The 3′ NTR of the HCV genome was identified as the primary HCV PAMP region that activates RIG-I signaling [54]. Importantly, this region is critical for HCV replication [55–58] and consists of three parts: a variable region containing two stem loops, a poly-U/UC-rich region that is single stranded and of variable length from 30 to more than 100 nt, and a conserved “X” region, which contains three stem loops (Figure 3). These components of the HCV 3′ NTR are present in all viral genotypes. It was expected that dsRNA or RNA with secondary structure located within the HCV RNA 3′ NTR would be the primary HCV PAMP for RIG-I. However, neither the highly structured X region nor the variable region dsRNA motifs of the HCV genome can activate RIG-I signaling. Remarkably, the poly-U/UC region, in conjunction with the essential 5′ppp, were identified as the HCV PAMP that serves to define the HCV RNA as nonself through recognition by RIG-I [54]. Importantly, while the 5′ppp was found to be absolutely necessary for RIG-I recognition of HCV RNA, it was not sufficient but specifically required the second nonself element, the poly-U/UC domain or its replication intermediate (the poly-A/AG domain of the negative-strand RNA), for stable binding to RIG-I that drives the conformation change of the RD and innate immune signaling activation [46]. Thus, nonself recognition of RNA as a PAMP likely requires multiple motifs of recognition by RIG-I that marks an RNA substrate as nonself or as a PAMP ligand to specifically stimulate innate immunity.

RIG-I signaling activation upon PAMP engagement results in RIG-I interaction with the downstream adaptor protein, IFN-beta promoter stimulator 1 (IPS-1). RIG-I/IPS-1 binding is mediated by the CARDs of each protein, This CARD-CARD interaction takes place on intracellular membranes and is anchored by IPS-1, leading to recruitment of a large signaling complex. In hepatocytes, this IPS-1 “signalosome” drives the activation of IRF-3 and NF-*κ*B by the IKK and/or Tank-binding kinase 1 (TBK1), protein kinases, and associated signaling partners. The exact mechanism by which interaction of RIG-I with IPS-1 activates signal transduction through IPS-1 remains to be determined, especially how RIG-I effectively relocalizes to interact with IPS-1 membrane-associated signaling domains. Moreover, recent studies now show that NLRX1 and the C1q receptor, gC1qR, can impart negative regulation to IPS-1-dependent signaling, indicating that RIG-I signaling activation must overcome this negative regulation in order to impart innate immunity [59–61]. However, the mechanisms of this regulation and how RIG-I may dominate to drive positive signaling are not yet known. Moreover, recent studies also show that mitochondria dynamics change during virus infection to impart enhancement of RIG-I signaling, but how this influences HCV infection and immunity is not defined [62, 63].

## 6. Evasion of the RIG-I Pathway by HCV

Despite the fact that HCV RNA can trigger RIG-I signaling of antiviral innate immunity, approximately 70–80% of all HCV-infected patients become chronically infected. Several studies have revealed that HCV can effectively evade various arms of the innate immune programs that are induced during infection, and that these outcomes are linked with an overall adaptive immune deficit that supports persistent virus replication and chronic infection.

HCV has evolved to counteract the RIG-I pathway [64], and this regulation serves to establish the infected hepatocyte as a platform for the propagation of chronic HCV infection. HCV evasion of RIG-I is mediated by the essential viral protein, termed NS3/4A. NS3/4A is a complex of the NS3 and NS4A proteins of HCV and serves to process the HCV nonstructural proteins from the precursor HCV polyprotein. NS3 interacts with its cofactor, NS4A, to anchor the NS3/4A complex to intracellular membranes and to facilitate complete activation of the NS3 protease domain [65, 66]. During the early stages of HCV infection and viral RNA replication, NS3/4A accumulation supports the targeted cleavage of IPS-1 by the serine protease activity of the NS3/4A complex [67–69]. Cleavage of IPS-1 by NS3/4A takes place near the IPS-1 C-terminal transmembrane domain, thus revealing IPS-1 and the IPS-1 signalosome from their membrane substrate (see Figure 1(a)). It has been shown that at early hours of HCV infection, the hepatocytes were able to relocalize IRF-3 from the cytoplasm to the nucleus; however, once cleavage of IPS-1 by NS3/4A occurs, which usually can be detected 24 hours after infection, none of the cells were found with nuclear IRF-3 [67]. As a result, IPS-1 can no longer signal downstream to activate IRF-3 and NF-*κ*B, and the infected cell no longer produced IFN-beta nor expressed ISGs. IPS-1 cleavage by NS3/4A completely disrupts RLR signaling and serves to block signaling through the RIG-I pathway during HCV infection [67, 69–71]. Indeed, IPS-1 mutation at the cleavage motif or NS3/4A protease inhibitor restores the RIG-I signaling pathway to stimulating the ISG expression and limit HCV infection [67, 72]. Recent studies have demonstrated that IPS-1 oligomerization is required for activation of antiviral innate immune signaling. Thus, it appears that disrupting IPS-1 oligomerization through NS3/4A proteolysis could be a contributing mechanism of RIG-I signaling repression [73, 74]. NS3/4A has also been shown to cleave the Toll/interleukin-1 receptor/resistance domain-containing adaptor-inducing IFN (TRIF) protein, the signaling adaptor molecule for TLR3, to prevent TLR3-mediated antiviral signaling [75, 76]. While TRIF proteolysis by NS3/4A would render the infected cell refractory to TLR3 signaling after PAMP ligation, a role for this process *in vivo* remains to be demonstrated [77, 78]. Of note is that TLRs, including TLR3, are themselves ISGs and would be expected to be induced as a result of RIG-I signaling. In this sense, the TLR3 axis may represent an important amplification loop that drives and diversifies the antiviral innate immune response to HCV infection.

## 7. Future Prospective

Our current understanding of RIG-I regulation by HCV is that the viral NS3/4A protease is the major inhibitor of RIG-I signaling through its ability to target and cleave IPS-1. Of high interest is that NS3/4A protease inhibitors are currently under development as antiviral therapies for HCV [79]. We have found that these protease inhibitors not only block the maturation of HCV NS proteins, but also can block the ability of NS3/4A to cleave IPS-1 and TRIF to restore innate immune signaling in HCV-infected cells [75, 76]. These features of HCV protease inhibitors should offer new treatment options with the effect of suppressing viral replication while enhancing the innate immune response to infection. Moreover, these data provide a proof of concept that therapeutic strategies aimed at enhancing RIG-I signaling could prove beneficial in the clinic for treating chronic or even acute viral infections. In addition to suppressing virus infection directly, the innate immune response serves to drive further inflammatory responses and adaptive immune programs that ultimately control infection and provide long-lasting immunity against further viral challenge. In this case, it will be important to fully understand RIG-I immune regulation in the context of the global immune response with the goal of defining sites of immune interaction that can offer therapeutic benefit through the development and use of immune-modulator drugs to treat HCV and other viral infections.
